# Factors Associated With Deficient Housing Among Community-Living Older Adults in the United States

**DOI:** 10.1093/geroni/igab046.1836

**Published:** 2021-12-17

**Authors:** Safiyyah Okoye, Laura Samuel, Sarah Szanton, Jennifer Wolff

**Affiliations:** 1 Johns Hopkins University, Baltimore, Maryland, United States; 2 Johns Hopkins Bloomberg School of Public Health, Baltimore, Maryland, United States

## Abstract

Housing quality is a recognized social determinant of health. Qualitative evidence suggests the ability of older adults to maintain their homes is affected by the domains of financial resources, social environment, and functional abilities, but this conceptualization has not been tested quantitatively. This cross-sectional study examined associations between financial resources (indicated by socioeconomic characteristics: education, racial-status, annual income, financial hardship, Medicaid eligibility), social environment (living arrangement, social integration), and functional abilities (lower extremity performance, self-care disability, independent-living disability, homebound-status, dementia, depression) with deficient housing among 6,489 community-living adults ≥ 65 years participating in the nationally representative 2015 National Health and Aging Trends Study. Sampling weights accounted for study design and non-response. An estimated 9.2% (3.2 million) older Americans lived in housing with ≥1 deficiency (any peeling paint, evidence of pests, flooring in disrepair, broken windows, crumbling foundation, missing siding, or roof problems). In bivariate logistic regressions, factors from all three domains were associated with deficient housing. In a multivariable model that included all variables above and adjusted for age and sex, indicators of financial resources and social environment remained associated with deficient housing (including financial hardship, adjusted odds ratio (aOR)=1.48, 95% confidence interval (CI): 1.10,1.98; and living with non-spousal others versus alone, aOR=1.48; 95% CI:1.09, 2.03), whereas indicators of functional abilities did not. To ensure quality housing for all community-dwelling older adults, efforts that increase financial resources and further examine the role of social environment in deficient housing are needed.

